# Transcriptome Analysis Indicates Immune Responses against *Vibrio harveyi* in Chinese Tongue Sole (*Cynoglossus semilaevis*)

**DOI:** 10.3390/ani12091144

**Published:** 2022-04-29

**Authors:** Xianghui Zhang, Xiancai Hao, Wenxiu Ma, Tengfei Zhu, Zhihua Zhang, Qian Wang, Kaiqiang Liu, Changwei Shao, Hong-Yan Wang

**Affiliations:** 1 College of Marine Technology and Environment, Dalian Ocean University, Dalian 116023, China; xianghuiz642@163.com; 2 Key Lab of Sustainable Development of Marine Fisheries, Ministry of Agriculture and Rural Affairs, Yellow Sea Fisheries Research Institute, Chinese Academy of Fishery Sciences, Qingdao 266072, China; best_hxc@163.com (X.H.); mawenxiu121@163.com (W.M.); tengfei.zhu@outlook.com (T.Z.); zhangzh0115@163.com (Z.Z.); wangqian2014@ysfri.ac.cn (Q.W.); liukq@ysfri.ac.cn (K.L.); shaocw@ysfri.ac.cn (C.S.); 3 Laboratory for Marine Fisheries Science and Food Production Processes, Qingdao National Laboratory for Marine Science and Technology, Qingdao, 266071, China

**Keywords:** Chinese tongue sole, *Vibrio harveyi*, immune response, transcriptome

## Abstract

**Simple Summary:**

Limited understanding of molecular mechanisms of immune response constrains marine fish farming. Analyzing the dynamic gene expression profile of fish in response to pathogen infection is gaining interest. We analyzed the expression changes of the Chinese tongue sole kidney after *Vibrio harveyi* infection with a series of transcriptome data. Notably, we observed rapid up-regulation of IL-17, TNF and TLR signaling pathways, indicating treatment measures should be taken in the early stage after infection. We also found a close connection between the immune system and neuroendocrine system, which may be the new strategy to improve immune function. Our research provides insights into disease prevention and treatment in fish farming.

**Abstract:**

Pathogenic infection of fishes is an important constraining factor affecting marine aquaculture. Insufficient understanding of the molecular mechanisms has affected the diagnosis and corresponding treatment. Here, we reported the dynamic changes of gene expression patterns in the Chinese tongue sole kidney at 16 h, 48 h, 72 h and 96 h after *Vibrio harveyi* infection. In total, 366, 214, 115 and 238 differentially expressed genes were obtained from the 16 h−vs. −C, 48 h−vs. −C, 72 h−vs. −C and 96 h−vs. −C group comparisons, respectively. KEGG enrichment analysis revealed rapid up-regulation of several immune-related pathways, including IL-17, TNF and TLR signaling pathway. More importantly, time-series analyses of transcriptome showed that immune genes were specifically up-regulated in a short period of time and then decreased. The expression levels of chemokines increased after infection and reached a peak at 16 h. Specifically, Jak-STAT signaling pathway played a crucial role in the regulation during *Vibrio harveyi* infection. In the later stages of infection, genes in the neuroendocrine pathway, such as glucocorticoid-related genes, were activated in the kidney, indicating a close connection between the immune system and neuroendocrine system. Our dynamic transcriptome analyses provided profound insight into the gene expression profile and investigation of immunogenetic mechanisms of Chinese tongue sole.

## 1. Introduction

The immune system is an important host defense system against viruses, bacteria and other pathogens. Once pathogens invade the organism, the innate immune system makes an immediate nonspecific reaction to clear the infection [1]. The kidney of teleost fish is a key tissue in the immune system [2]. During activation of the immune system, pathogen-associated molecular patterns are recognized by pattern-recognition receptors (PRRs) of the innate immune system. Toll-like receptors (TLRs) are the most important class of PRRs that can specifically recognize various pathogens and activate immune signaling [3]. After pathogen recognition, TLRs stimulate rapid activation of innate immunity by inducing the production of proinflammatory cytokines and the up-regulation of costimulatory molecules [4]. Studies on the kidney of Japanese flounder (*Paralichthys olivaceus*) proved the expression changes of TLRs and interferons (IFNs) after vaccination against viral hemorrhagic septicemia virus (VHSV) [5]. Several studies have indicated that the kidney secretes several types of hormones, such as glucocorticoids (GCs) and cortisol, and plays important roles in immune systems [6,7,8]. Therefore, the analysis of immune-neuroendocrine connections is crucial to elucidate the function of kidneys during the immune reactions [9,10].

In the immune response, multiple cytokines function synergistically, including interleukins (ILs), chemokines, IFNs and transforming growth factor (TGF) [11,12]. ILs have diverse physiological functions, and their regulatory mechanism in the inflammatory process in teleost fish is conserved [13]. The intraperitoneal injection of IL-1β in rainbow trout (*Oncorhynchus mykiss*) increases the number of phagocytes that migrate into the peritoneal cavity and the activity of lysozyme in macrophages [14]. The administration of ILs with vaccines also enhances antibody production, which suggests that ILs may be exploited as an immune adjuvant for improving vaccine efficacy [15]. Chemokines are inflammatory mediators needed to clear pathogens during an immune reaction. The excessive release of chemokines is the major cause of hyper-inflammation [16]. Furthermore, the cytokines in the immune system interact with the neuroendocrine system to maintain homeostasis in an organism. Numerous studies have shown that cytokines (such as TNF, IL1, IL6 and IL10) can stimulate the hypothalamic-pituitary-adrenal (HPA) axis and kidney to induce the release of glucocorticoids, which are important for the immune response [17,18,19,20]. Subsequently, glucocorticoids influence the viability and proliferation of monocytes, the migration of lymphocytes, and the production of proinflammatory mediators [21,22].

Chinese tongue sole is a valuable marine aquatic species distributed in northern China. However, with the rapid development of intensive agriculture, disease outbreaks occur more frequently and are costly to the aquaculture industry. *Vibrio harveyi*, a gram-negative bacterium is one of the main causes of infection in Chinese tongue soles. The infection is associated with the high mortality rate of the fish, accompanied by surface ulcers and erosion of the tail. *V. harveyi* is the major reason for *Luminous vibriosis*, which affects a diverse range of marine animals, including penaeids [23], bivalves [24] and teleost [25]. *V. harveyi* was highly pathogenic to Atlantic salmon and rainbow trout, with mortalities of up to one hundred percent after intraperitoneal injections (10^6^ cells/fish) [26]. Furthermore, the use of antibiotics has been restricted due to residual and resistant pathogens. Therefore, the most effective strategy to prevent disease outbreaks in fish aquaculture is to improve the immune function of fish. In this case, we analyzed the response pattern of the immune system after *V. harveyi* infection with transcriptome analysis, which could offer new insights for studying the immune response of Chinese tongue soles.

## 2. Materials and Methods

### 2.1. Experimental Fish Treatment and Sample Collection

Chinese tongue soles with a bodyweight of 438.4 ± 53.0 g and a body length of 41.0 ± 2.7 cm were obtained from Laizhou Ming Bo Aquatic Co., Ltd. (Laizhou City, China). The Chinese tongue soles were temporarily housed in seawater at a temperature of 20 °C for 48 h. The fish were then intraperitoneally injected with the same concentration of *V. harveyi* (1 × 10^4^ CFU (colony forming unit)/mL) based on their weight (4 μL/g). All fish were anesthetized with MS-222 before sample collection. The fish were sampled at 0 h, 16 h, 48 h, 72 h and 96 h post-infection treatment, and the kidneys were collected from three individual fish at each time point and frozen in liquid nitrogen immediately. Then, the tissues were stored at −80 °C until RNA extraction.

### 2.2. RNA Extraction and Library Sequencing

TRIzol reagent (Invitrogen, USA) was used to extract total RNA following the manufacturer’s instructions. A Qubit RNA Assay Kit with a Qubit 2.0 Fluorimeter (Invitrogen, USA) was used to quantify RNA, and the RNA integrity was then evaluated using an RNA Nano 6000 Assay Kit and a Bioanalyzer 2100 system (Agilent Technologies, Santa Clara, CA, USA). In total, 15 cDNA libraries were constructed with a TruSeq Stranded mRNA LT Sample Prep Kit (Illumina, San Diego, CA, USA) according to the manufacturer’s instructions. Each library was then sequenced on an Illumina NovaSeq 6000.

### 2.3. Data Filtering and Genome Mapping

The quality of the raw reads of 15 samples was assessed using FastQC. Low-quality reads, reads with adaptors and too many N were filtered with SOAPnuke [27]. The clean reads were mapped to the Chinese tongue sole genome using HISAT2 v2.1.0 [28]. Subsequently, salmon_smem was used to calculate the expression levels of each gene based on estimated transcripts per kilobase million (TPM). Differentially expressed genes (DEGs) between different groups were determined using DEseq2 v3.14, and the parameters of a false discovery rate (FDR/q-value) < 0.05 and |log_2_FoldChange| ≥ 1 were used to define the DEGs [29].

### 2.4. Comprehensive Analysis of DEGs 

Functional annotation analysis and enrichment analysis of DEGs were performed through Gene Ontology (GO) and Kyoto Encyclopedia of Genes and Genomes (KEGG) analyses. The phyper package of R software was used to enrich the analyses based on the annotations of DEGs obtained from the GO and KEGG analyses (*p*-value < 0.05). The protein-protein interaction networks were constructed using String v10 and visualized the hub biological interactions among genes using Cytoscape v3.8.2 [30]. Mfuzz was used to reveal expression patterns of DEGs over time (v2.52; Options = -c 4, -m 1.25) [31]. The heatmaps were generated to show gene expression levels with the R package Pheatmap v1.0.12.

### 2.5. Quantitative Real-Time PCR Validation

To further validate the reliability of the RNA-seq data, immune-related DEGs were selected for qRT-PCR verification. The primers used in the present study are listed in Table 1. qRT-PCR was performed with ABI StepOnePlus Real-Time PCR System (Applied Biosystems, USA) and SYBR quantitative kit (Qiagen, Hilden, Germany). The methods and reaction systems were in accordance with the instructions. The reaction procedure was as follows: 95 °C for 2 min; 40 cycles of 95 °C for 5 s and 60 °C for 10 s; 95 °C for 15 s, 60 °C for 1 min, +1 °C/min; and 95 °C for 15 s. β-actin was used as reference (*β-actin*-qF/qR, Table 1). Three replicates of each reaction system were included in the study, and the relative expression of the genes was analyzed using the 2^−ΔΔCT^ method.

## 3. Results

### 3.1. Transcriptome Sequencing Data

To understand the immune mechanisms of Chinese tongue sole kidney in response to *V. harveyi* infection, we performed transcriptome sequencing of a total of 15 samples, including four infected and one control groups. As shown in Supplementary Table S1, we obtained a total of 85.96 GB of clean data from 15 samples, and average of about 5.73 GB of clean data was generated for each sample. Afterward, the clean reads were mapped to the Chinese tongue sole genome [32], and the average mapping rate was 91.76%. The RNA-seq data was deposited in the CNGB under accession number CNP0002542.

### 3.2. Gene Expression during V. harveyi Infection

**Figure 1:**
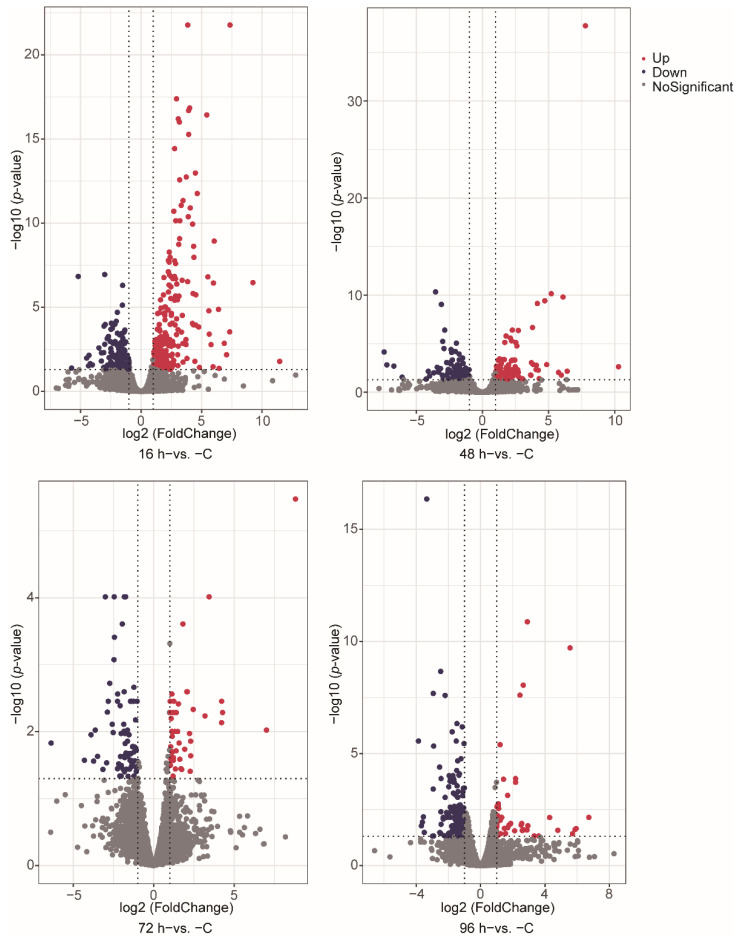
Differential expression analysis of genes between different stages. (**A**–**D**) The volcano plot shows the differential expression genes, including 16 h−vs. −C (**A**), 48 h−vs. −C (**B**), 72 h−vs. −C (**C**) and 96 h−vs. −C (**D**). The red and dark purple colors indicate up- and down-regulated DEGs in the different group comparisons, respectively.

**Figure 2:**
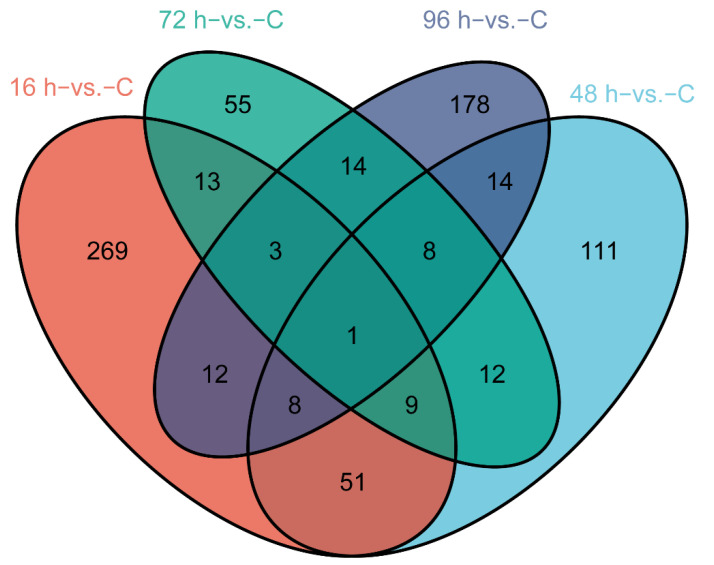
Venn diagram showing the DEGs in the four groups and the shared DEGs among the comparisons.

We identified DEGs involved in *V. harveyi* infection of fish at different stages after infection compared with normal fish (*p*-value < 0.05, fold change ≥ 2). The numbers of significantly up- and down-regulated genes were as follows: 245/121 genes in the 16 h−vs. −C comparison, 100/114 genes in the 48 h−vs. −C comparison, 43/72 genes in the 72 h−vs. −C comparison and 43/195 genes in the 96 h−vs. −C comparison (Figure 1A–D). A Venn diagram was used to show the total and shared DEGs among the different comparisons (Figure 2). Just the ryanodine receptor 3 (*ryr3*) was identified among the four groups.

### 3.3. GO and KEGG Functional Enrichment of the DEGs

**Figure 3:**
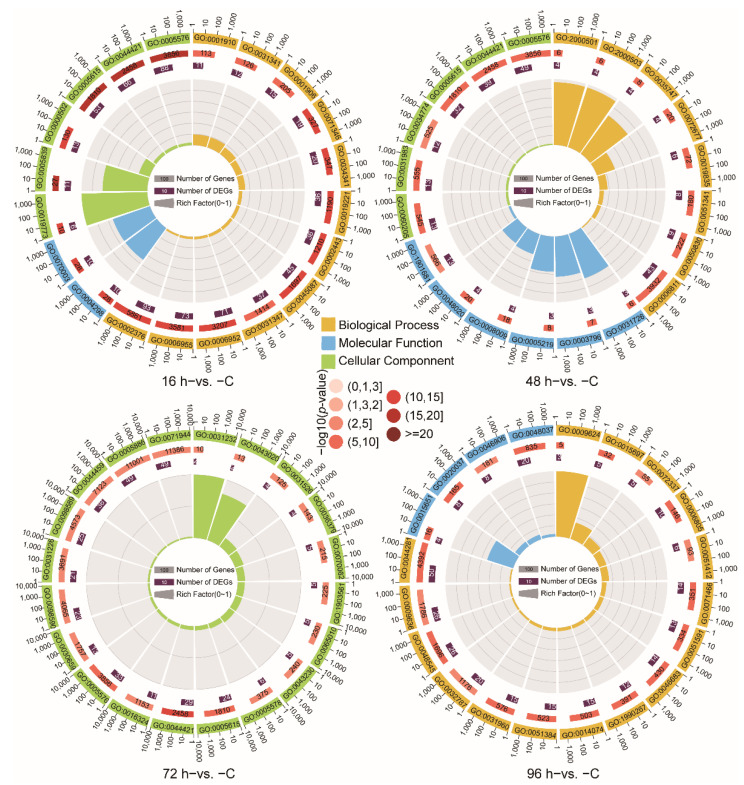
Top 20 terms in the GO functional classification of DEGs identified from the different group comparisons (*p*-value < 0.05).

To further reveal the immune response of the Chinese tongue sole against *V. harveyi* infection, the thematic associations of the DEGs, GO and KEGG enrichment analyses were performed. A GO term enrichment analysis was conducted, and 1424, 1217, 877 and 1045 GO terms were obtained from the 16 h−vs. −C, 48 h−vs. −C, 72 h−vs. −C and 96 h−vs. −C comparison groups (*p*-value < 0.05), respectively. In the 16 h−vs. −C, 48 h−vs. −C and 72 h−vs. −C comparison groups, the most enriched GO terms were Extracellular space (GO:0005615) and Extracellular region (GO:0005576) (*p-*value < 0.05). In addition, enrichment of the Defense response (GO:0006952) and Immune response (GO:0006955) in the 16 h−vs. −C comparison and Defense response to Gram-positive bacterium (GO:0050830) and Natural killer cell chemotaxis (GO:0035747) in the 48 h−vs. −C comparison were observed. In the 72 h−vs. −C comparison, the enriched GO terms included Extracellular exosome (GO:0070062) and Extracellular vesicle (GO:1903561). The GO terms Xenobiotic metabolic process (GO:0006805), Response to glucocorticoid (GO:0051384) were enriched in the 96 h−vs. −C comparison (Figure 3, Supplementary Table S2).

**Figure 4:**
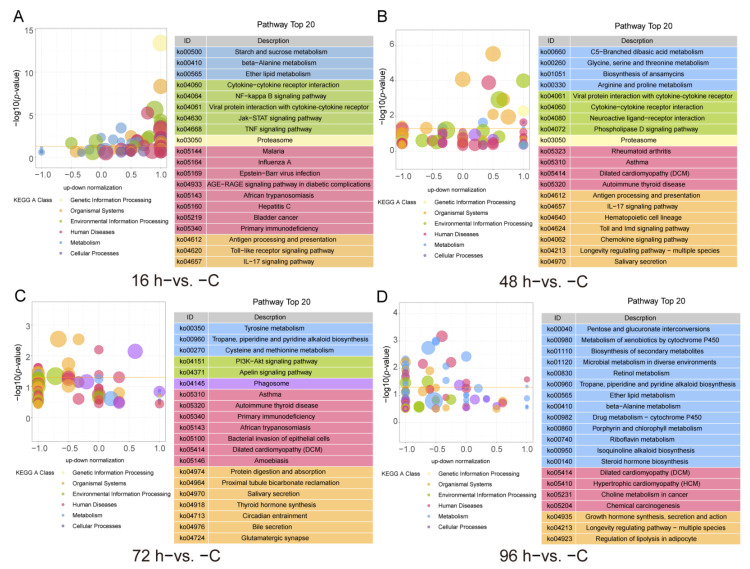
Top 20 pathways in the KEGG functional classification of DEGs identified from the different group comparisons (*p*-value < 0.05), 16 h−vs. −C (**A**), 48 h−vs. −C (**B**), 72 h−vs. −C (**C**) and 96 h−vs. −C (**D**).

The KEGG pathway enrichment analysis results, which were shown in Figure 4, revealed that 41, 16, 15 and 30 pathways were significantly enriched in the 16 h−vs. −C, 48 h−vs. −C, 72 h−vs. −C and 96 h−vs. −C comparison groups (*p*-value < 0.05), respectively. In the 16 h−vs. −C comparison, the enriched KEGG immune pathways mainly included Antigen processing and presentation, Cytokine-cytokine receptor interaction, IL-17 signaling pathway, NF-κB signaling pathway as well as the jak-STAT signaling pathway and TNF signaling pathway (Figure 4A, Supplementary Table S3). The enriched KEGG pathways in the 48 h−vs. −C comparison were the IL-17 signaling pathway, Toll and imd signaling pathway, Antigen processing and presentation, Chemokine signaling pathway and Cytokine-cytokine receptor interaction (Figure 4B, Supplementary Table S3). In the 72 h−vs. −C comparison, the KEGG pathways identified included Tyrosine metabolism, Phagosome, PI3K-Akt signaling pathway and Thyroid hormone synthesis (Figure 4C, Supplementary Table S3). The KEGG pathways identified in the 96 h−vs. −C comparison included the Biosynthesis of secondary metabolism, Metabolism of xenobiotics by cytochrome P450, Retinol metabolism and Growth hormone synthesis, secretion and action (Figure 4D, Supplementary Table S3).

### 3.4. Time-Series Expression Profile of DEGs during V. harveyi Infection

**Figure 5:**
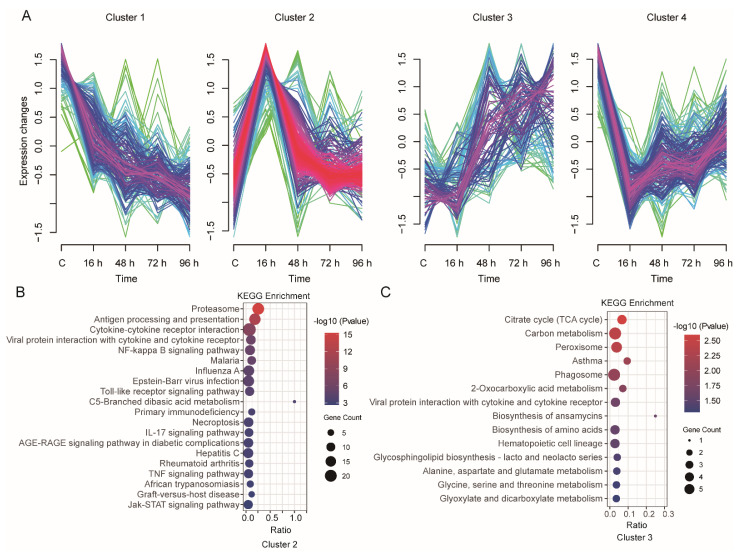
Transcriptome-wide time-series cluster of DEGs. (**A**) Cluster analysis of DEGs based on Mfuzz. (**B**,**C**) Functional categorization of DEGs in Cluster 2 ((**B**), *n* = 270) or Cluster 3 ((**C**), *n* = 117) by KEGG analysis.

In total, 758 DEGs were identified from the four group comparisons (Supplementary Table S4), and these DEGs were further analyzed using Mfuzz to profile their time-series expression after infection. These clusters exhibited genes with specific expression patterns and were classified into four clusters (Figure 5A). The genes in Cluster 1 (177 genes) showed that the expression levels were decreased after infection. Cluster 2 (270 genes) was the most abundant cluster, and the included genes showed an increase in expression at 16 h after infection followed by a decrease; the genes in Cluster 3 (117 genes) showed a decrease at 16 h and then sharply increased at 48 h after infection; the genes in Cluster 4 (194 genes) showed the opposite expression pattern to those in Cluster 2, with decreased expression at 16 h followed by an increase in expression.

We further investigated the function of these DEGs from 4 clusters through GO and KEGG pathway enrichment analyses (Supplementary Table S5). The genes in Cluster 1, Cluster 2, Cluster 3 and Cluster 4 were significantly enriched in 17, 53, 19 and 15 KEGG pathways, respectively (*p*-value < 0.05). The KEGG pathways in Cluster 2 were mainly related to the immune system, such as the Antigen processing and presentation, Cytokine-cytokine receptor interaction, NF-κB signaling pathway, Toll-like receptor signaling pathway, IL-17 signaling pathway as well as TNF signaling pathway and Jak-STAT signaling pathway (Figure 5B). In Cluster 3, the main enriched pathways were metabolism-related pathways, such as Carbon metabolism and Glycine, serine and threonine metabolism. We also focused on Viral protein interactions with cytokine and cytokine receptor pathways (Figure 5C).

### 3.5. Key Immune-Related DEGs and Protein-Protein Interaction Networks

**Figure 6:**
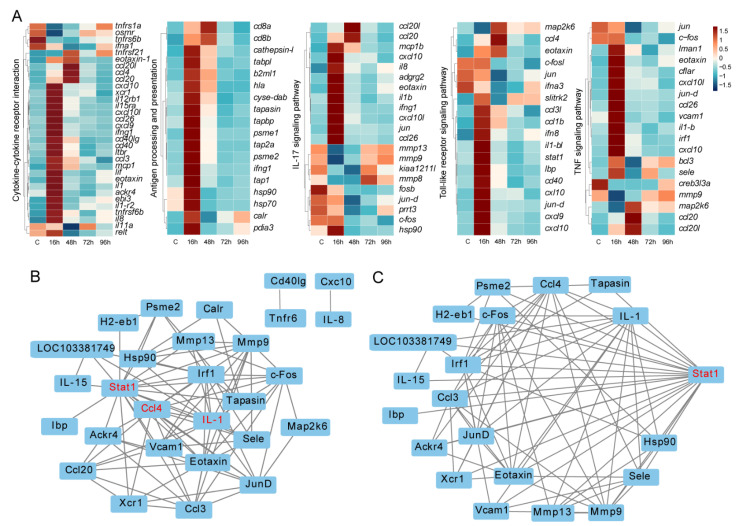
Expression patterns and protein-protein interaction networks of DEGs. (**A**) Expression patterns of DEGs in immune-related pathways. (**B**) Protein-protein interaction networks of the immune-related proteins. (**C**) Relationships between Stat1 and other selected immune-related proteins.

Based on the GO and KEGG functional enrichment analysis results, many immune-related signaling pathways were enriched. We focused on 5 KEGG immune pathways, including Cytokine-cytokine receptor interaction, Antigen processing and presentation, IL-17 signaling pathway, Toll-like receptor signaling pathway and TNF signaling pathway. A total of 72 DEGs directly involved in immune responses were found in all infected groups compared to controls. Results showed the rapid response of immune genes at the early stage. Interestingly, the expression levels of responding genes decreased subsequently (Figure 6A).

To reveal the relationships of these proteins, a protein-protein interaction (PPI) network analysis was conducted (Figure 6B). Among these, 28 proteins had strong expression correlations, such as Stat1, Ccl4 and IL-1, which were assumed to be hub proteins that play significant roles in the immune response (Figure 6B). Specifically, the relationships between Stat1 and other proteins are presented in Figure 6C.

### 3.6. Validation of DEGs by qRT-PCR

**Figure 7:**
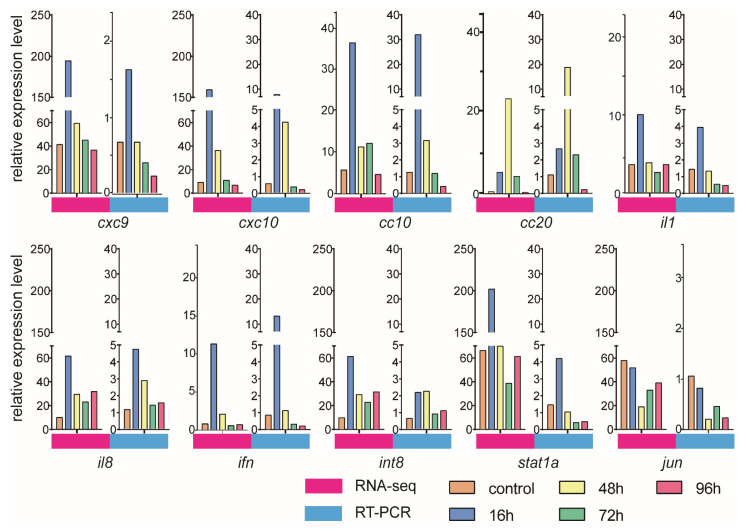
Expression levels of immune genes verified by both qRT-PCR and RNA-seq.

To validate the gene expression dynamics in response to *V. harveyi* infection, we selected immune genes from RNA-seq data for qRT-PCR analysis. The qRT-PCR and RNA-seq results were consistent, as shown Figure 7. Although the measured gene expression patterns were slightly different from those obtained from the transcriptome analysis, the trends were basically the same. For example, the *cxc9*, *cxc10* and *cc10* expression levels increased after infection, reached a peak at 16 h and then decreased sharply.

## 4. Discussion

Chinese tongue sole is an economically and nutritionally important aquaculture species in northern China, and bacterial pathogens can cause disease outbreaks and inflict substantial economic damage in farming [33]. *V. harveyi* has proven to be a highly pathogenic pathogen in marine fish in recent years [34]. However, the potential molecular mechanism of the immune response against *V. harveyi* infection in Chinese tongue sole remains poorly understood. Previous studies have focused more on the comparison of infected fish with healthy ones [35]. Few is known about the gene expression dynamics and the regulation mechanisms of immunity within a certain period of time after infection. In this study, we infected the Chinese tongue sole with *V.* *harveyi* for the time period from 16 to 96 h. Transcriptome analysis was carried out to analyze the gene expression signatures and potential regulatory mechanisms in response to infection. Thus, our research may provide a framework for understanding the molecular pathogenesis and immune defense responses in the Chinese tongue sole.

As vital defenders against invading pathogens, TLRs are essential in innate immunity. After recognizing pathogens, TLRs activate innate immunity rapidly and stimulate the production of proinflammatory cytokines such as ILs, IFNs, TNF and chemokines, which are crucial for eliminating pathogens [36,37]. IL-1, which is generated by macrophages after activation, can regulate the immune response and promote antibody production by B lymphocytes [38]. IL-8 is extremely important in immune defense because it coordinates the activities of lymphocytes and macrophages and participates in the acute inflammatory response [39]. Furthermore, IFNs and chemokines are also important classes of proinflammatory cytokines that are generally considered to maintain immune homeostasis, modulate inflammatory responses and perform other immune functions. During *V. harveyi* infection, a large number of innate immune pathways were found to be up-regulated in the kidney, and these included the IL17 pathways, cytokine-cytokine receptor interaction and Antigen processing and presentation, which were enriched in the 16 h−vs. −C and 48 h−vs. −C group comparisons. The up-regulated expression of proinflammatory cytokines suggested that the proinflammatory response may be an important antibacterial mechanism at the early stage of infection. Moreover, chemokines play a crucial role in the process after infection. CCLs and CCRs interacted with many immune-related genes, including ILs, CD genes and TLRs in the immune response [40]. In our data, the expression levels of both chemokines and some ILs were increased at 16 h after infection. The dynamic patterns of the gene expression level detected by both RNA-seq and qRT-PCR are similar. These results indicated the network of these immune genes after infection and provided insights for teleost disease defense. 

In mammals, the Jak-STAT pathway plays critical roles in the initiation and regulation of innate immune responses through signaling [41]. The *stat1* gene is vital to the effectiveness of the Jak-STAT pathway and potentially to antibacterial activity in fish [42,43]. In our study, the Jak-STAT signaling pathway was enriched in Cluster 2. During infection, the *stat1* gene was up-regulated at 16 h, and protein-protein interaction analysis indicated core roles for the Stat1 protein in an immune-related pathway. This finding indicates that Jak-STAT may promote innate immune processes and anti-inflammatory functions by mediating an immune pathway that plays a critical immune function during the infection of the Chinese tongue sole with *V. harveyi*. The activation of ILs, TNF, TLRs, IFNs and chemokines was found to be beneficial for innate immunity and essential for the clearance of *V. harveyi* in Chinese tongue sole, and many of the related pathways were associated with the Jak-STAT signaling pathway. Therefore, targeting the Jak-STAT pathway could be a new approach for improving immunity in the Chinese tongue sole in the future.

The teleost fish kidney is not only an immune organ but also a key mediator of the immune-neuroendocrine system. Consequently, accumulating evidence suggests that anti-inflammatory hormones, such as glucocorticoids, can stimulate and influence the immune response [2,44,45,46]. Our DEGs enrichment analysis showed that the GO term Response to glucocorticoids was enriched at 96 h (96 h−vs. −C comparisons) (Figure 3). And the KEGG enrichment analysis also identified Thyroid hormone synthesis at 72 h (72 h−vs. −C comparisons) (Figure 4). The exploration of glucocorticoid-immune interactions shows that GCs appear to be potent immunosuppressive agents with complex actions in the immune responses of fish [37,47]. In mammals, GCs inhibit the NF-κB pathway and the synthesis of ILs and IFNs, leading to decreased production of proinflammatory cytokines [48]. During immunosuppression in fish, cold-shock and heat-shock stress induce reductions in the levels of complement components and the enrichment of GCs [49]. Furthermore, the heat shock protein (HSP) interacts with GCs to regulate proinflammatory and anti-inflammatory cytokine expression [50]. In the present study, we also found that *hsp90* showed differential expression in response to *V. harveyi* infection (Figure 5A). Therefore, GCs and HSP may be immune adjuvants that improve the immune system of fish.

The KEGG pathway enrichment analysis also found that Retinol metabolism was enriched in the 96 h−vs. −C comparison, and in the immune system, retinol can increase the immunoglobulin levels through effects on B lymphocytes [51]. Furthermore, our KEGG enrichment analysis of Cluster 3 showed that many metabolic pathways were enriched. These results indicated that in late infection, the immune system also affects the neuroendocrine system in Chinese tongue sole infected with *V. harveyi*. 

## 5. Conclusions

In conclusion, we successfully studied the infection of the Chinese tongue sole with *V. harveyi* and revealed changes in immune-related genes in the kidneys. Our results identified immune- and endocrine-related DEGs at different infection stages. We found that various immune pathways, such as those involving ILs, TNF, TLRs, cytokines, apoptosis, phagocytosis or metabolism, were significantly correlated with pathogen infection. We also discovered that glucocorticoids, HSP and Retinol metabolism were involved in the immune reaction, which indicated that the kidneys can impact the neuroendocrine and metabolic systems.

## Figures and Tables

**Table 1:** Specific primers used for qRT-PCR.

Prime	5′–3′
*ifn*-qF	CTTGTCAGGTCTTGACCCTG
*ifn*-qR	GTAACAGCAGGTTTTGGATGG
*cxc-9*-qF	CAGCAAAGTGACAGTGAT
*cxc-9*-qR	AGGCACACTTTCACATCA
*cxc-10*-qF	CAAGTCAGAAGGTGTCAG
*cxc-10*-qR	GGAAGTGACTGGAGTTGG
*int-8*-qF	TGAAGAACTGAAACTGCAACACT
*int-8*-qR	TGCTGATCGGTACTATTCCATTG
*cc10*-qF	CCAGAGTCACCACTTGGAAA
*cc10*-qR	GCTGAGGTTCCTGAGTTTGTT
*cc20*-qF	GTTTCAGGTGATCAAGGGCT
*cc20*-qR	TCGTCCCTCTTAGTCACACA
*il8*-qF	ACCGATCAGCAGGGACTTTA
*il8*-qR	CTTCTTCCCGTTCACCAGAC
*jun*-qF	TTTCTCCCAGCACGAAAACA
*jun*-qR	GGGATGTAAGGATGTCGCTC
*il1*-qF	GGACATCATCTGCACAACCA
*il1*-qR	TAGAGGCATACGACACCAGT
*stat1*-qF	TCACTAAACGGGGCCTAAAC
*stat1*-qR	CTTTCTCGTTAGCACTCTGCTT
*β-actin*-qF	GCTGTGCTGTCCCTGTA
*β-actin*-qR	GAGTAGCCACGCTCTGTC

## Data Availability

The raw sequencing data are available through the CNGB data accession number CNP0002542.

## Supplementary Materials

The following supporting information can be downloaded at: https://www.mdpi.com/article/10.3390/ani12091144/s1. Table S1: Summary statistics for the sequencing data of each sample. Each infection stage included three biological repeats. Table S2: The GO terms of different group comparisons. Table S3: The KEGG pathways of different group comparisons Table S4: The differential expression genes of 758. Table S5: The KEGG pathways of Cluster 1, 2, 3 and 4.
