# Supplementary material for: Transcriptome Analysis Indicates Immune Responses against Vibrio harveyi in Chinese Tongue Sole (Cynoglossus semilaevis)
Source: Animals (Basel). 2022 Apr 29;12(9):1144. doi: 10.3390/ani12091144 (PMC9104532; doi:10.3390/ani12091144)
Supplement: Supplementary file 1 [file animals-12-01144-s001.zip › animals-1651411-supplementary Table S1.pdf]

Table S1. Summary statistics for the sequencing data of each sample. Each infection stage included three biological repeats.

Sample	Raw Reads	Clean Reads	Mapping rate (%)	Q20 (%)	Q30 (%)	GC Content (%)
16 h-1	48,744,342	47,380,914	89.41	97.63	93.61	45.45
16 h-2	20,170,075	18,834,413	91.54	97.69	93.68	46.66
16 h-3	21,022,086	19,803,598	93.10	97.80	93.94	48.02
48 h-1	14,757,791	14,057,000	92.68	97.75	93.81	47.67
48 h-2	18,762,036	17,765,218	92.17	97.86	94.07	47.30
48 h-3	26,146,200	24,587,372	89.01	97.82	94.02	46.37
72 h-1	25,150,483	23,499,457	91.35	97.81	93.94	47.25
72 h-2	23,622,199	22,011,400	90.69	97.48	93.08	46.65
72 h-3	22,146,806	20,901,235	92.68	97.80	93.93	47.98
96 h-1	23,899,889	22,505,519	91.87	97.76	93.86	47.36
96 h-2	22,921,104	21,622,031	92.74	97.72	93.80	47.83
96 h-3	21,379,701	20,218,361	92.94	97.60	93.49	48.05
C-1	24,834,930	23,603,597	92.38	97.84	94.02	47.98
C-2	24,900,444	23,413,354	92.79	97.81	93.96	47.98
C-3	20,765,102	19,536,779	91.01	98.00	94.41	47.12
Summary	359,223,188	339,740,248	91.76	97.76	93.84	47.31
